# Demand-driven capacity building for public health nutrition research in Lao PDR

**DOI:** 10.1186/s41256-024-00378-7

**Published:** 2024-09-18

**Authors:** Gerald Shively, Ramya Ambikapathi, Kate Eddens, Susmita Ghosh, Nilupa S. Gunaratna, Kelley Khamphouxay, Ratthiphone Oula, Kethmany Ratsavong, Thipphakesone Saylath, Latsamy Siengsounthone, Patricia Sipes, Vanphanom Sychareun, Carmen Tekwe, Leah Thompson, Souksamone Thongmixay, Maikho Vongxay, Viengnakhone Vongxay, Roger Zoh

**Affiliations:** 1 Purdue University, 615 West State St., West Lafayette, IN 47907 USA; 2 Cornell University, Ithaca, NY USA; 3 Indiana University, Bloomington, IN USA; 4 Catholic Relief Services, Vientiane, Lao PDR; 5 Lao Tropical and Public Health Institute, Vientiane, Lao PDR; 6 University of Health Sciences, Vientiane, Lao PDR; 7 Nutrition Center, Vientiane, Lao PDR

**Keywords:** Institutional strengthening, Higher education, Nutrition, Public health, Research

## Abstract

In Laos, rates of undernutrition, especially among children under 5 years of age, remain high. In response, a large multidisciplinary team embarked on a multi-year project in Laos beginning in 2019 with the purpose of institutional strengthening around public health nutrition research. This paper summarizes the Applied Nutrition Research Capacity Building project’s activities, immediate project results, and prospects for sustaining impacts into the future. Eight primary activities were undertaken, including back-office strengthening, mentored research, and curriculum review and development. Requested training and skill development in areas related to public health nutrition, anthropometry, and research methods reached more than 1000 professionals. The first edition of a Lao-English Nutrition Glossary was produced, as was the country’s first National Nutrition Research Agenda, a document which sets locally-identified priorities for future research. Project success was achieved by focusing on the priorities of project partners and the Lao government, as articulated in the Lao National Nutrition Strategy and Action Plan. Project design elements that could guide similar efforts undertaken elsewhere include multi-year engagement, an emphasis on sustained peer mentorship, and the use of an extended period of pre-planning in collaboration with project stakeholders prior to the start of activities.

## Background

Despite sustained international efforts to eradicate undernutrition, it remains the primary health threat to young children [1, 2]. In 2022, 149 million children below age five worldwide were stunted (low height-for-age) and 45 million were wasted (low weight-for-height), only slightly fewer than in 2018 [1, 3]. Undernutrition is disproportionately prevalent and has the greatest disease burden in low- and middle-income countries [4]. Undernourished children are at a higher risk of death from common illnesses such as diarrhea, pneumonia and malaria [5], and face greater risk of impairments in intellectual performance, work capacity and lifetime health and earnings—critical impediments for a country’s economic growth and development. Undernourishment in childhood also perpetuates a cycle of malnutrition, as malnourished women face greater odds of giving birth to malnourished, low-birth-weight infants when they reach reproductive age [1].

In the Lao People’s Democratic Republic (henceforth, Laos), rates of malnutrition, especially among children under five years of age, remain high. The most recent data reveal that 33% of Lao children below age five are stunted, 11% are wasted and 24% are underweight, with higher percentages among some sub-groups and relatively little improvement over the past five years [6, 7]. In response to ongoing concerns about these undesirably high rates of child malnutrition, the Lao government has identified as a long-term objective the establishment of a National Institute of Nutrition (NIN). Such an institute would, ideally, provide a nationwide and interdisciplinary focus on public health nutrition, and effectively channel investments by the Lao government and the donor community into building the nation’s capacity to conduct local and culturally-sensitive research, translate research findings into action, and communicate goals and activities to a diverse set of stakeholders in the Lao community and beyond.

In 2019, with support from the U.S. Agency for International Development (USAID), a multidisciplinary team implemented a multi-year project to support institutional strengthening for public health nutrition research in Laos. This paper describes the Applied Nutrition Research Capacity Building (ANRCB) project’s activities, which were undertaken to strengthen capacity to conduct and utilize nutrition research. We review immediate results achieved by the project as well as prospects for sustained impacts. The project incorporated design elements that can inform and guide similar and future efforts undertaken in other countries.

## Project objectives

### Identifying capacity building as the primary project goal

*Capacity building* refers to efforts targeted at strengthening and enhancing the ability of an individual, organization or community to perform effectively in a particular domain. Capacity building tends to address specialized management issues, and often involves developing the knowledge, skills, resources and infrastructure necessary to achieve specific goals and objectives [8]. For low- and middle-income countries facing significant food and nutrition security challenges, specifically-targeted capacity building combined with effective nutrition governance can be fundamental to achieving improved nutrition outcomes [9]. As in other countries, building this capacity in Laos has been difficult given competing demands for government resources and shifting donor priorities. As a result, constraints on research capacity have produced a cascade of effects in which the local production and application of contextually-relevant problem-solving research is difficult. As in many settings, this dynamic presents a major challenge to improving public health.

The overarching goal of the ANRCB project was to improve capacity to conduct and utilize nutrition research, incorporating four key components of capacity building: (1) human resources development; (2) infrastructure enhancement; (3) knowledge and skill transfer through targeted training, mentoring and “hands-on” experiential learning; and (4) institutional collaboration and strengthening. The project targeted three key Lao institutions with responsibility for public health nutrition programming and training: the Ministry of Health’s Nutrition Center (henceforth referred to as the Center), the University of Health Sciences (UHS), and the Lao Tropical and Public Health Institute (Lao TPHI). Two US-based academic institutions—Purdue University and Indiana University—provided technical support, and Catholic Relief Services (CRS), which has sustained a strong physical presence in Laos and the South East Asian region generally, served as the in-country implementing partner. Purdue University is home to a highly-regarded College of Agriculture and Department of Nutrition Science. Indiana University has a global reputation in public health education. Importantly, both institutions have researchers who are internationally known for their global field work and training in nutrition and epidemiology, agricultural economics and food policy, and public health. CRS brought a track record of working successfully with the Lao government and engaging in capacity building activities with multiple stakeholder groups in Laos. The core team consisted of individuals with many years of international project experience in South East Asia, Africa and Latin America, which contributed to project success. Past experiences often informed not only project design, but the ability to continually and adaptively manage the project, incorporating local knowledge and needs along the way, a hallmark of effective project management [10].

### Implementing a results-oriented framework

To begin, the project identified a set of fundamental requirements for success: (1) a clear understanding regarding the individuals and institutions with which we would work, including their roles and responsibilities; (2) a well-articulated problem/solution analysis; (3) an appreciation of the needs of partners and stakeholders as articulated by them and those with whom they were interacting; (4) a desire for sustainable change among partners; and (5) feasibility of activities within the Lao operating context and implementation period.

## Project design and implementation

### Phased implementation

**Fig. 1:**
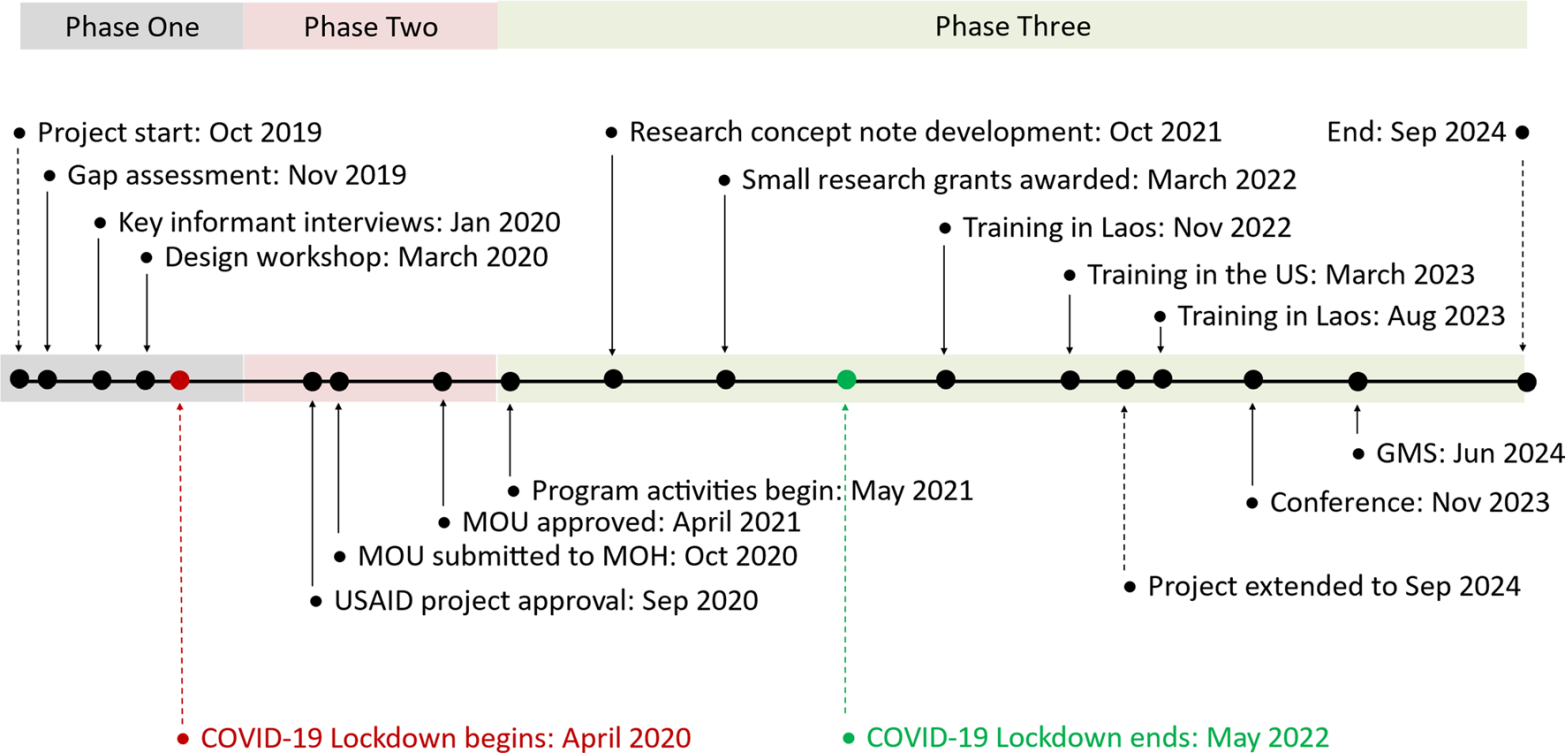
Timeline of project phases, activities and events

The project was designed and implemented in three phases, as illustrated in Fig. 1. Phase 1 began in September 2019 and consisted of eight months of discovery and pre-planning, including a site visit by US-based investigators, who conducted key informant interviews with more than 100 government and international non-governmental organization (INGO) stakeholders in Laos, and an assessment (through structured interviews and surveys) of basic knowledge, skill gaps, and training needs among those with whom we would work, including government staff, university faculty, students, and recent graduates. These activities were designed to orient the team to the existing environment for nutrition activities in Laos and the priorities of the government within the context of the Lao National Nutrition Strategy and Action Plan (NNSAP) [11]. The potential receptiveness of Ministry of Health (MoH) leadership and staff to training and capacity building was also assessed.

Phase 2 consisted of twelve months of activities undertaken in the US and Laos to (1) design, develop and translate research and training materials; (2) work with stakeholder audiences to position the project for wide visibility; and (3) work with Center staff to increase basic institutional capacity in areas such as office operations, communication and financial management. This long project runway was both necessary and useful because we were also developing a Memorandum of Understanding (MOU) with the government of Laos and seeking government approval for project implementation. Phase 3, consisting of both remote and in-country activities, began once the MOU was approved and signed. These activities took place over twenty-six months from May 2021 through September 2024.^1^

### Using data and information to drive project design

Before identifying, defining, and budgeting for a set of project activities, we engaged in a gap assessment and co-creation process during Phase 1.^2^ Criteria for identifying gaps that could be relevant for the project’s focus included: (1) need for attention; (2) government openness to receiving outside assistance; (3) lack of redundancy or competition with other ongoing donor-led interventions; (4) feasibility to introduce activities and achieve results and short-term impacts within the project life; and (5) reasonable likelihood of sustained post-project efforts. Motivated by the interdisciplinary nature of public health nutrition, activities included building institutional infrastructure and mentorship support; strengthening research capacity in an interdisciplinary way; and establishing and maintaining communication toolkits to rapidly disseminate results to a range of stakeholders.

To design project activities, we used information from a range of assessments to better understand core needs and potential constraints and challenges to implementation. Among the assessment methodologies and tools used were structured conversations with stakeholders, literature reviews, key informant interviews, and online and face-to-face structured surveys. Specifically, we engaged in the following:

- A scoping visit by the US team to identify potential activities. This visit included a full week of meetings with sponsor staff, representatives of the Lao government, university representatives and staff of various INGOs working in the nutrition space.
- A literature review to understand the institutional and programmatic context of nutrition in Laos, as well as local research and scientific capacity. Materials reviewed included data (such as Lao Multi-Indicator Cluster Survey data), Lao and external agency reports, various unpublished INGO/donor research reports, and literature covering government policy, strategy, and organization. The latter included the Lao National Nutrition Strategy to 2025 and Action Plan 2016–2020 [12], an unpublished midterm Review of the Nutrition Action Plan, and unpublished documents from the 2019 National Nutrition Forum, including the National Progress Report of 2019 [13].
- Key Informant interviews with more than 45 individuals, including both Lao and external stakeholders, which was synthesized to maintain confidentiality of participants. Sensitivity was regarded as important to ensure candid responses on topics that could be construed as critical of current practices, in recognition that community involvement in study design and implementation would be essential for project success [14–16].

Building on findings from the scoping work listed above, structured survey instruments were developed and anonymous surveys were conducted to collect information specific to individuals’ nutrition knowledge, training, and work responsibilities. Information included both quantitative and qualitative data. Surveys were developed in English and then translated into Lao.^3^ Surveys were administered in Lao using both on-line and face-to-face methods, and responses were collected from 27 university faculty and students and 22 members of leadership and staff of the Center. Characteristics of survey respondents are summarized in the Appendix. As we developed training activities and materials in Phase 2 of the project, we used information obtained through the survey to help ensure materials were sensitive to the gender, backgrounds, and needs of participants.

Once survey data had been reviewed, the team met over two days to map findings to key themes, summarize answers to key questions, build a problem/solution tree, determine which problems/solutions were already being addressed by other stakeholders and which problems/solutions would be feasible for the project to address, and, finally, brainstorm possible project points of entry. Organizational charts were developed and vetted with partners and stakeholders to better understand institutional staffing structures and relations among government offices. Led by these insights, we identified five key areas in which the project could provide support to our partners.

*Area 1: Emphasizing the role of research in policy design*. At project inception, a relatively small number of professionals in Laos, university faculty included, had had the opportunity to receive formal research training or develop skills related to publishing research in international outlets or competing for grants. As a result, efforts to conduct public health and nutrition research had been severely hampered. We identified a desire to better coordinate and communicate with governmental and non-governmental stakeholders, and a desire on the part of university staff to better link their research to policy needs.

*Area 2: Upgrading technical skills.* Many academics and professional staff expressed a strong desire to upgrade their skills through formal training in technical nutrition and research topics. The limited number of Lao researchers who had received formal training on public health nutrition research was seen as undermining mentoring of the next generation of Lao researchers. We identified strengthening of the local research community as a way to reinforce interest in pursuing and maintaining research careers.

*Area 3: Improving institutional infrastructure*. The Nutrition Center had been established relatively recently in relation to the project, in 2012. Research infrastructure and management systems in place at the Center were still evolving, which provided the project with an opportunity to help support their desire to be a convener of research, work more efficiently across ministries, and help translate research findings into policy guidance for the government. Cross-ministerial cooperation was identified as a challenge. Leadership expressed an understanding of the importance of collaboration and convening relevant ministries but had not functioned as a convener or developed ways to gather research and then translate findings into evidence-based policies. The Center also asked for assistance to support day-to-day management and financial systems to work more efficiently and collaboratively, and to ensure quality standards were being met.

*Area 4: Meeting international norms.* Providing support for training and tools for nutrition researchers and staff at the Center and UHS was seen as a way to help these groups to improve the quality of their work and help them to meet international norms, with the understanding that such norms are not often clearly articulated. Attention focused on research ethics, professional writing, and the use of citation management software. The project also sought to identify and fill gaps in the nutrition curriculum at the university level and improve research methods in university and government settings. Whenever possible and appropriate, the project emphasized hands-on training to allow learners to put new skills into practice, with mentorship and follow-up to track quality of learning.

*Area 5: Enhancing communication and dissemination of information to the community and policymakers.* The project developed approaches to assist and support communication of nutrition research information and findings. Coordination was encouraged between various actors involved in nutrition across the health, education and agriculture sectors to help reduce information silos. The project worked to support new avenues (for Laos) of research communication, for example monthly webinars, a quarterly newsletter, research posters, and participation in national and regional conferences. To engage the small and nascent Lao private sector the project worked with the SUN Business Network in Laos in four ways. One, we invited private companies to respond to our nutrition stakeholder survey. Two, we put these companies on our newsletter distribution list. Three, we made sure the national health IRB committee invited private sector research firms to a seminar on how to apply for ethical approval. And four, we consistently shared all relevant project materials with the SUN Business focal point and invited them to project training activities.

### Developing a strategy for capacity building

The conclusion of Phase 1 resulted in our planned strategy for building capacity, which focused on undertaking two sets of activities: (1) training and capacity building for the Center to conduct research, with particular focus on helping to create a sub-unit “Center of Excellence” in anthropometrics within the Center; and (2) training and research experiences within UHS and Lao TPHI to support and foster curriculum improvements and updates, and to promote an overall research-friendly environment and evidence-based approach to addressing nutrition challenges. In addition, a cross-cutting theme was to work continually with the Center, the MoH, allied ministries, and other nutrition stakeholders to create institutional and communication structures that could be sustained after the conclusion of the project.

The project design was informed by the Lao National Nutrition Strategy and Action Plan (NNSAP) [11], as well as a draft Action Plan for 2021–2025. In designing the project, the team studied the NNSAP in detail and discussed likely points of collaboration with government and non-government stakeholders. The design was also informed by an Organizational Capacity Development Plan for the Center prepared before the project by an outside consultant. Because many Center staff were appointees who had not had opportunities to receive formal training in nutrition or public health research, the project aimed to provide basic nutrition knowledge, skills related to nutrition assessment (especially anthropometric measurement) and field research, interpretation of research findings, and capacity to inform policy. Key stakeholders identified at the start included specific groups within university and INGO communities.^4^ In developing a capacity building plan, we paid particular attention to the following questions: (1) What are the key issues and problems facing the nutrition sector? (2) What critical assumptions underpin our approach, and how are these (e.g., budgets, language) linked to factors that would influence our design choices? (3) What proof of concept might lead the GoL to sustain project activities? And (4) What might be the exit/phase-over preferences of the government?

After considering these questions and assessment information, we identified activities the project could feasibly undertake based on several criteria, including: Is the activity feasible based on institutional and political features in Laos? Can the project influence the outcome? Are any other groups or organizations engaged in the activity? Is the project likely to achieve results and foster improvements given the time and resources available?

### Ensuring collaborative and participatory design

In March 2020, roughly four months after the start of the project, the team conducted a week-long design workshop in Vientiane. The intent was to conduct the workshop with all project participants present in Vientiane but due to the pandemic, the workshop was held in a hybrid format, with those from Purdue and IU participating remotely from the US and those from CRS (and occasional invited guests) participating in Laos. The aim was to (1) establish project goals; (2) identify and refine proposed activities and sub-activities; (3) identify responsible parties; and (4) develop an implementation plan, timeline and budget for activities. The workshop was informed by the November site visit and the extensive efforts undertaken during the ensuing period to identify and meet with as many Center stakeholders as possible. Key individuals with in-depth experience working in Laos were brought in to discuss context and to answer clarifying questions. Daily notes were logged and, in some cases, individuals external to the project were asked to review these notes and provide reactions. This “closed loop” approach was used to brainstorm strategies for mapping from areas expressed by stakeholders as critically in need of support to strategic objectives that could guide the design of project activities.

A primary conclusion reached during the design workshop was that among all possible strategic partners for the project, the MoH and the Center were the best candidates. The MoH, as the convener of the Lao Nutrition Secretariat, provided a natural and formal connection to other ministries represented in the Secretariat (i.e., Ministry of Agriculture and Forestry, Ministry of Planning and Investment, and Ministry of Education and Sports). We also identified UHS and Lao TPHI as strategic higher education institution (HEI) partners. As a risk-management strategy, we deliberately identified a set of independent activities to be pursued separately with the Center and the HEIs, to guard against unforeseen barriers or constraints that might limit progress with any particular group and thereby jeopardize overall project momentum and success. That said, the plan involved coordinating training activities to include Center, UHS and Lao TPHI staff and students. The idea was that this would not only economize on team efforts but also help enhance the working relationship between government and universities, which was a strategic aim for the project.

### Mapping needs to implementation

**Fig. 2:**
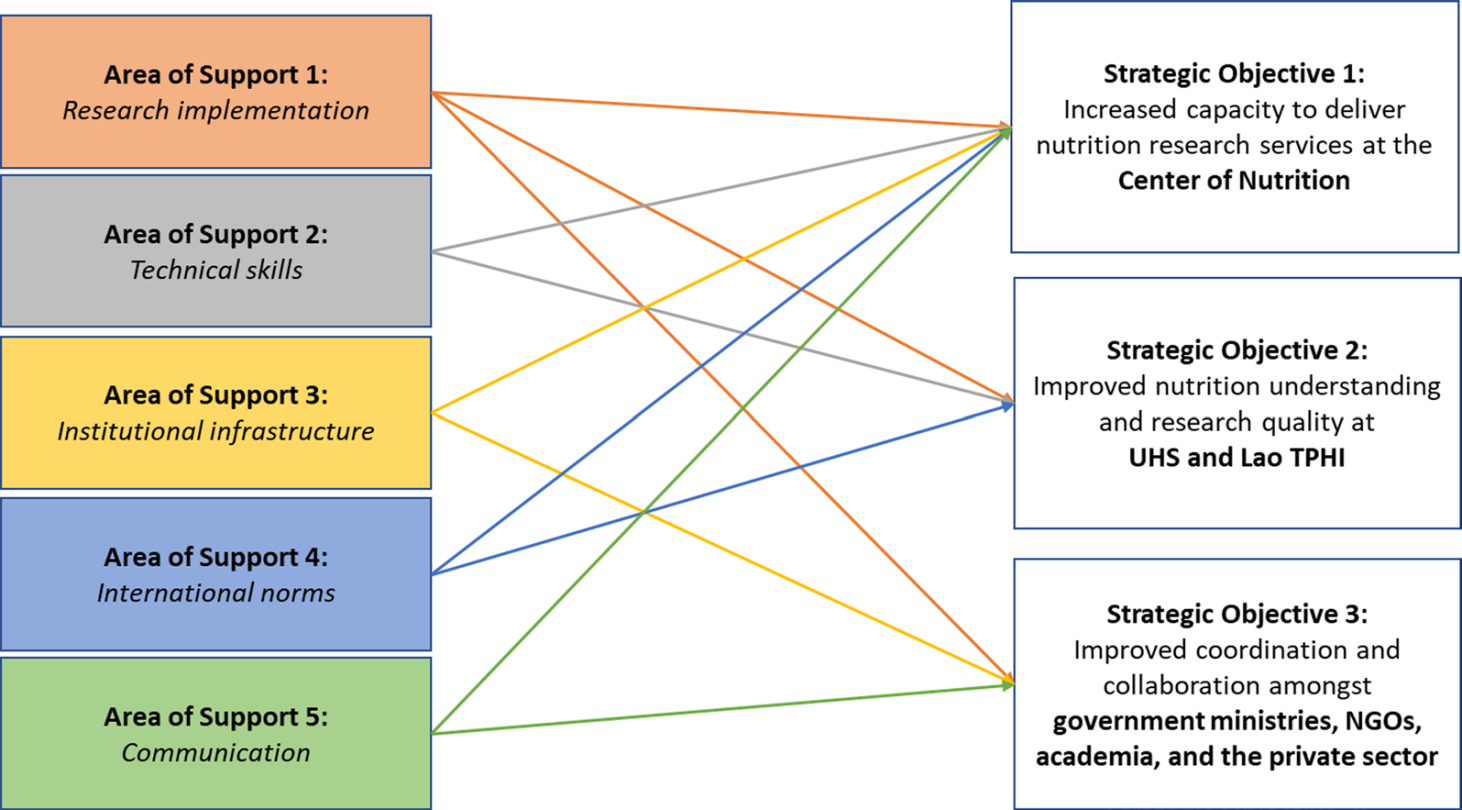
Primary mapping from areas of support to strategic objectives

A primary outcome of the design workshop was a proposed implementation plan. During the workshop, the five key Areas of Support were mapped to three Strategic Objectives, i.e., the most ambitious results the project could hope to achieve (see Fig. 2).

From the Strategic Objectives, nine domains for project activities were identified. These constituted the practical steps to be undertaken by the project to generate the desired changes in target knowledge, behaviors, and actions. Table 1 lists the five areas of support, along with the project response and examples of specific activities undertaken by the project. Strategic Objective 3 was largely treated as a cross-cutting issue and was supported through efforts to include all groups and stakeholders in project activities whenever possible.

**Table 1:** Key areas of support

Area of support	Project response	Embedding demand from lao scientists	Examples of activities
1. Implementing research and emphasizing the value of empirical research	Joint activities involving university researchers and Ministry of Health staff	UHS and Lao TPHI scientists identified and prioritized research and training topics	Monthly webinars, regional study exchanges, collaborative research and training, development of a National Nutrition Research Agenda
2. Upgrading technical skills	In-person and on-line short courses	Lao team identified child malnutrition as a key priority	Training in anthropometry, basic and advanced statistics, survey design, data handling, etc
3. Improving institutional infrastructure	Back-office training and mentoring at the Center	Targeted training was provided on budgeting for project proposals	Financial reporting, facilities and human resources management, scheduling systems
4. Adhering to international norms and benchmarks for research	Short-term training and peer-to-peer mentoring as part of small research grants	Project support for participation in regional and international conferences and training in Thailand and the United States	Research ethics, institutional review board (IRB) approval, citation management, writing workshops, participation in regional and international conferences
5. Enhancing communication and dissemination of activities and findings to the community and policymakers	Printed and online materials and collaboration with dissemination networks such as SUN business network	The team identified a lack of consistency when translating nutrition terms from English to Lao	Dual-language nutrition glossary, dual-language quarterly newsletter, monthly webinars with simultaneous translation

After identifying key indicators, the project established a system for monitoring, evaluation, adaptation, and improvement based on results and feedback [17]. The system prioritized collection of feedback and data, as well as listening to the voices of the partners and stakeholders. The monitoring and evaluation system allowed rapid adaptive management to address any signs of ineffectiveness or misplaced effort.

## Project achievements^5^

### Activity 1: “Back Office” system strengthening

CRS worked closely with the Center from the start of the project to provide training and coaching on financial management, gender and social inclusion (GESI) integration, project management, and staff onboarding. These key areas responded to an institutional needs assessment conducted before the project. This type of institutional accompaniment is frequently neglected in projects, but is essential to support operational effectiveness and increase program quality, which in turn increases the sustainability of all other programming and provides value to all stakeholders who rely on institutional strength to support program-critical activities. To facilitate, a formal hosting agreement was signed, which allowed project staff to sit across the hall from Center staff, promoting daily interaction to build relationships of trust and support. Quarterly partnership meetings with the Center were used to provide regular opportunities to assess knowledge and behavior change, celebrate success, and engage Center leadership in supporting system strengthening changes.

### Activity 2: Developing physical space for training

A prior project constructed a complex of buildings as a development assistance package to the MoH. Our team assisted in transforming empty space into a facility for group training and co-working, including large and small training rooms for multiple uses and a small library. In September 2021, the Center held an opening ceremony attended by the Lao Minister of Health and the US Ambassador. In addition to equipping the physical space, the project worked with the Center to establish protocols for maintenance and upkeep of facilities and equipment.

### Activity 3: Strengthening capacity and skills for anthropometric assessment

Given the Lao government’s stated interest in addressing child malnutrition, and the strong interest in anthropometry among Center leadership, the project engaged in extensive training for anthropometric data collection and assessment, as well as oversight to create and maintain facilities, equipment, and expertise to sustain accurate anthropometric measurement. The training modalities being employed at the Center were not fully known at the start of the project, but our conversations during Phase 1 with a wide range and large number of stakeholders, including Center staff, suggested room and desire for improvement. We also recognized that once data collection modalities had been strengthened, capacity building in data entry and analysis would be essential for staff to analyze and accurately interpret Lao indicators.

### Activity 4: Curricula review at the Lao University of Health Sciences (UHS)

Anticipating that many project training activities would be undertaken at UHS, early in the project we asked university leaders to identify interests and needs, and to provide a comprehensive review of programs and existing curricula. Prior to the project, UHS had a track record of training students in a Masters of Public Health (MPH) program. During early project scoping, it became clear that the MPH program placed little emphasis on nutrition. Developing in students a more holistic understanding of nutrition across the life course was seen as beneficial by university leadership. Course program descriptions and syllabi (some available in English and some translated from Lao) were reviewed for content and teaching methods. This resulted in a review report containing suggested additions and examples of program designs at other institutions. It also guided the development of project training modules which were later used to augment existing course materials to fill gaps in teaching materials.

### Activity 5: Short-term training

Short-term training was a centerpiece of the project. Over the course of the project, subject matter experts worked with Lao partners to develop twelve, multi-part video lessons, consisting of more than 40 h of content. These modules, and their intended audiences, are listed in Table 2. Some modules were specifically developed for staff at the Center and some were developed with UHS and Lao TPHI staff and students in mind. Nearly all included pre- and post-training assessments and teaching guides in both English and Lao.

**Table 2:** Training modules and target audiences

Topic	Target audience
Basic nutrition concepts and terms	Nutrition Center staff; undergraduate students
Nutrition assessment methods	Nutrition Center staff; UHS and Lao TPHI faculty and graduate students
Research concepts	UHS and Lao TPHI faculty and graduate students
Anthropometry—children	Nutrition Center staff; UHS and Lao TPHI faculty and graduate students
Anthropometry—adults	Nutrition Center staff; UHS and Lao TPHI faculty and graduate students
Anthropometry—advanced topics	Nutrition Center staff; UHS and Lao TPHI faculty and graduate students
Food Environments	Nutrition Center staff; UHS and Lao TPHI faculty and graduate students
Food safety	Nutrition Center staff; UHS and Lao TPHI faculty and graduate students
Behavior change	Nutrition Center staff; UHS and Lao TPHI faculty and graduate students
Research design and planning	UHS and Lao TPHI faculty and graduate students
Statistical analysis	UHS and Lao TPHI faculty and graduate students
Academic writing	UHS and Lao TPHI faculty and graduate students

Training topics directly targeted at university instructors and students included modules on research design, research methods, research coordination and priority setting. Modules focused on practical activities and strategies for converting knowledge to action. The project worked with UHS and Lao TPHI faculty to support uptake and classroom use of modules in degree programs, and to identify training needs and gaps. For example, to identify gaps in current scientific knowledge and strengthen a basic understanding of food safety concepts, the Purdue team completed a background review on food safety issues in Laos to inform the development of a training module. Training sessions frequently combined participants from the Center, UHS and Lao TPHI, enhancing interaction and networking between these groups.

Throughout the project, more than 60 group training sessions were conducted with more than 1000 participants. Certificates of completion were issued to those who completed trainings. Many in the target audience participated in single trainings, while others participated in nearly all. In total, the project reached more than 400 unique individuals, including professional staff, university faculty, and students. A general working principle when developing training modules was to begin by assessing Lao institutional demand as a driver, emphasizing the importance of examples, especially Lao examples, and promoting group-based exercises and activities to capitalize on local learning styles and modalities. Wherever possible and appropriate, modules were developed in collaboration with Lao partners, first in English and then translated into Lao. Group discussions and activities facilitated learning among participants with varying proficiency in English. Content was disseminated through local partners and a project YouTube channel. Modules were used in a number of ways, including synchronous in-person and on-line sessions, as well as asynchronous self-guided sessions.

**Fig. 3:**
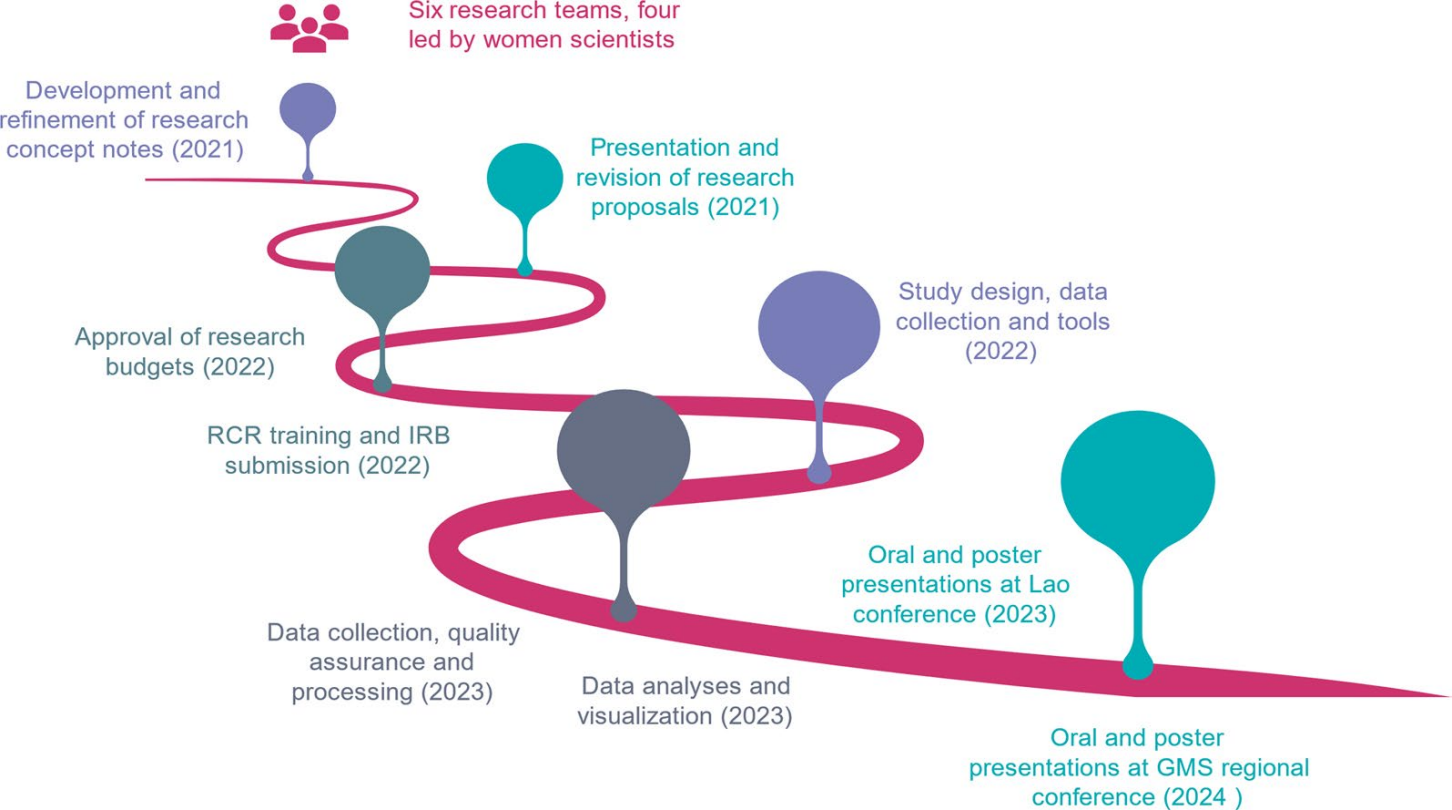
Sequence of steps used for the small research grants (SRGs)

In addition to topic-based training, the project incorporated team-based research activities. During Phases 2, a “Small Research Grant” (SRG) program was introduced. The goal was to provide a guided, funded, and mentored research experience through the sequence of steps outlined in Fig. 3. Each team consisted of 4–6 researchers from the Center, UHS or Lao TPHI, and each team was paired at the start of the process with mentors from Purdue, Indiana or Cornell universities. Teams worked with their mentors by email and through virtual meetings, and also benefited from multiple reciprocal visits to each other’s institutions, including 4–6 week stays in the U.S. by SRG leaders during the final writing stage. Laptops and statistical software were provided to each team. Sequencing of the SRG steps was scaffolded to relevant training modules, for example assessment methods, research study design, statistical analysis, and academic writing. Whenever possible, these hands-on, closely monitored and mentored research collaborations targeted junior faculty and young researchers in Laos, thereby helping to enhance capacity within the respective Lao institutions to provide and sustain mentoring and scientific good practices into the future.

### Activity 6: Lao-English nutrition glossary

The team identified a lack of consistency when translating nutrition terms from English to Lao. Many terms, especially technical terms, could only be translated with difficulty, and resources such as Google’s translate feature were often unreliable in rendering correct translations. In response, the team worked with the Center and university researchers to identify key terms, develop the most accurate and appropriate translations, and generate a glossary to ensure proper and consistent translation across stakeholders of nutrition and nutrition research in Lao. This activity was used as a practicum to support the Center in putting into practice project management skills, and the MoH fully embraced the project, eventually producing 4,000 copies of the glossary for distribution in schools, clinics and health facilities throughout the country.

### Activity 7: Enhanced communication

To increase multi-sectoral cooperation and awareness of nutrition research in Laos, a communication strategy was developed that centered around outputs that were new, and hence innovative, for our Lao colleagues: a quarterly newsletter highlighting nutrition research being conducted in-country, and a locally-led seminar/webinar series. Both channels were designed to highlight recent and ongoing research. The newsletter included links to key resources, advice, and opportunities, as well as meeting announcements. It was produced in both Lao and English and distributed via email, WhatsApp, and various social media channels to key stakeholders in government, INGOs, civil society organizations, and academia. Webinars, which included real-time translation, were launched with research presentations from project staff but evolved to include non-project researchers. Becuase both activities were new in the Lao context, assigning Center staff in leadership positions provided an opportunity to build Center capacity in multi-sectoral communication and convening. Although these functioned as one-way forms of communication, topics were chosen based on feedback from our Lao partners and, over time, local experts played a greater role in providing the content.

### Activity 8: National nutrition research agenda

An over-reliance on donor-led research can reduce in-country agency for evidence-based policy-making, creating unpredictable research cycles and fragmentations in the health system [18]. In 2016, Lao TPHI produced a summary of nutrition and health research topics and, in 2018, developed a National Health Research Agenda with 11 priority topics. However, at the start of the project, Laos did not have a research agenda for nutrition. To address the need for such a document, the project engaged with researchers at Lao TPHI to design a process to identify research gaps and needs for nutrition research in Laos, and prioritize research needs. From the start, the goal was to ensure local ownership and control of identifying and prioritizing nutrition research gaps in Laos. The priority-setting exercise included national-level policymakers (e.g., from the MoH), the National Nutrition Committee (representing multiple sectors), the Mother and Child Health Center (MCHC), and local members of the NGO and INGO communities. Interviews were conducted with more than 30 policymakers from a range of sectors, including nutrition and health researchers and practitioners. The aim was to explore perceived research needs using these key informant interviews. Simultaneous to this, a literature review was conducted to collect published findings of relevance to the nutrition situation in the country. Subsequent review and listening sessions narrowed the list of topics to eight primary themes and 68 sub-themes. These were then incorporated into a survey administered to 160 stakeholders, including district and provincial health officers, provincial hospital staff, and members of the Ministry of Health’s National Nutrition Committee. Participants ranked topics in terms of importance, and the resulting ranking was used to develop a prioritized list of 60 research questions. These were further vetted with senior stakeholders and published in early 2024 as the *National Nutrition Research Agenda 2023–2026* (NNRA). The NNRA now serves as the first guiding document for nutrition research in Laos.

## Policy implications and lessons learned

### Addressing the need for local ownership

In many nutrition policy discussions, there is a well-recognized need for local, context-specific evidence, as well as development and coherence across local institutions and stakeholders. This project provides an example of how to address this need, in a demand-driven way that enables local ownership and does not erode agency in setting the research agenda. True capacity building takes time, resources, and human leadership. Emphasizing quality over quantity as well as consistency of engagement over time is essential to building capacity. Despite being classified as a low-middle-income country, Laos still grapples with a weak health system, both in terms of physical infrastructure and human capital [17]. Going forward, understanding the national context of needs and developing local ownership of challenges and their solutions will be critical for setting priorities (for example, as seen in the development of the NNRA) which can then be targeted for improvement across various dimensions of work. Three specific aspects of local ownership stand out.

First, empowering individuals and embracing a diversity of experiences is key. To sustain impact from capacity building efforts, researchers need to be equipped to work in multidisciplinary teams to collaborate effectively. This requires strong research teams and partnerships with institutions that share similar goals, philosophies, and a willingness to embrace local empowerment. For this project, our target training audience represented multiple disciplines, which required our team to sometimes rethink our approach to nutrition research and adjust trainings accordingly, especially to cover areas of knowledge not previously encountered. For example, many professionals in our target audience had been trained in medicine, not nutrition or research. In this project's context, however, diversity of experience and training often deepened conversations and created shared understanding among those with different perspectives. Fostering community among researchers through training across institutions, creating opportunities for networking, and supporting engagement in national, regional and international scientific conferences help individuals to benefit from diverse perspectives and experiences.

Second, given the large number of Lao ethnicities and languages, wherever possible, tools and materials used should be adapted to and validated to local contexts and languages. This ensures relevance and effectiveness in training activities and also ensures knowledge translation through effective communication and dissemination of research findings among stakeholders, including policymakers, researchers, and communities. An acknowledged shortcoming of our project is that while “local ownership” of activities means Lao ownership, not all groups were represented in the project and many areas of indigenous knowledge and practice were not included. In the future, as the Lao community of public health nutrition researchers continues to grow in number and capacity, it will be important to ensure that research findings are translated into actionable policies and interventions to achieve sustainable improvement in local systems. For example, Laos still lacks country-specific dietary guidelines, food-based recommendation guidelines, laboratories for nutrient analysis, and national food composition tables. So long as the country relies on resources borrowed or adapted from neighboring countries, it will be difficult to ensure that the country’s nutrition challenges are being fully embraced and “owned.” Local ownership is especially important in higher education, where curriculum improvements and teaching enhancements require absolute sensitivity to local norms and practices. Interventions using evidence from research must be responsive to local needs, as the use of appropriate tools, materials and methods can improve knowledge and enhance the skills of professional staff [19]. Closing the loop on local adaptation and validation requires a monitoring and evaluation system that supports adaptive management so that feedback from end-users can be used to adjust programs and practices.

Third, independent governance and funding of research teams can help provide a greater sense of ownership and encourage young researchers to build research careers in-country. This can be achieved when governments allocate specific budgets for research and implementation with transparency and using methods that reward effectiveness and accountability. When activities are supported in ways that encourage collaboration among a range of stakeholders, including researchers, implementation teams, and policymakers, the process of translating research findings into actionable policies can be accelerated.

### Challenges to local ownership

Multiple challenges to fostering local ownership exist, especially in the realm of addressing nutrition and food security challenges, not least because addressing malnutrition comprehensively requires navigating the co-existence of different forms of malnutrition, including undernutrition and—increasingly—overnutrition. Stakeholder groups may have sometimes opposing or conflicting goals or perspectives. In a country like Laos, with considerable ethnic diversity, linguistic and geographic barriers pose challenges to reaching vulnerable populations, especially in remote areas. Infrastructure limitations, such as poor road conditions and lack of basic amenities, further exacerbate challenges. Limited English proficiency can hamper access to information and undermine collaboration, which means effective communication strategies are needed to bridge the gap between researchers, policymakers, and communities. A project such as the ANRCB, constrained by institutional forces in its geographic reach and focusing by necessity on capacity building in key institutions, was limited in its ability to work beyond the capital region and the Lao-English language nexus.

Often, dependency on foreign funding undermines local budget allocations for health and nutrition research. This can exacerbate weaknesses in monitoring and evaluation within the health system. In addition, where various entities, including NGOs, universities, and various government agencies operate independently and in an uncoordinated fashion, efforts can overlap, sharing of findings can be difficult, and assigning ownership as well as monitoring and evaluation can be difficult. A project design that intentionally brings these groups together can help to build better working relationships.

Finally, “brain drain” is a significant and recognized challenge. It takes two forms. The familiar one arises when trained and highly-skilled individuals leave the country. But an equally pernicious version arises when trained individuals leave their positions, e.g., in government or higher education, for local jobs in unrelated but more remunerative settings. Both types of brain drain undermine capacity building efforts. Engaging in joint-research (see, e.g., [20]) can help strengthen the credentials, not just the skills, of local researchers, and reinforce commitment to a career path. The ANRCB project provides an example of ways that local talent can be provided with opportunities for personal and professional growth, which can help strengthen career commitment and open doors to professional opportunities.

### Potential strategies to achieve local ownership

As with most externally-funded and externally-led projects, achieving local ownership has been a central goal as well as an ongoing challenge for the ANRCB project. Local ownership of project activities and outputs can occur through multiple approaches, some of which have gained more traction than others. Important aspects that allowed us to promote some degree of local ownership include first and foremost having government entities as primary partners and signing our MOU with the Ministry of Health, which ensured high-level attention, regular feedback, and project accountability to government priorities. Strategies employed to engage stakeholder audiences and involve them in project design and implementation include the use of various modes and methods of interaction, including online and in-person trainings and learning labs; in-person and hybrid seminars, webinars, and workshops; direct collaboration with universities to build capacity effectively and prioritize university needs surrounding curriculum development and training needs; and working with Lao partners to draw lessons from research conducted in different countries but similar contexts to inform Lao approaches. Helping to support scientific research conducted in Laos has meant that classroom examples can be more relevant and engaging for students, reflect local contexts, and meet the needs of end-users, which should help enhance ownership over project resources.

Incentivizing professional growth was a central aim of the project, and was achieved by providing paired, long-term mentorship and goal setting for staff through initiatives such as financial support for research and short-term residencies as visiting scholars, English language training and training certification. The project organized an in-person “watch party” for the 2023 Agriculture, Nutrition, and Health (ANH) Academy, the first of its kind for Laos. The project also co-sponsored a major Lao public health conference in November 2023, at which a panel discussion and poster session were used to highlight and disseminate the work of the project and Lao project partners. Major involvement in and sponsorship of the 14th Greater Mekong Subregion (GMS) Public Health Conference in June 2024 also provided an opportunity to showcase project output and promote regional research networking with academics and practitioners from more than 20 academic institutions from Cambodia, China, Laos, Myanmar, Thailand, Vietnam, the United States, the Netherlands and Australia. As an innovation for the conference, the project team worked with the conference organizers to identify topics and conduct a series of eight, half-day pre-conference “learning lab” workshops, which attracted the participation of more than 400 conference attendees.

Such immersive approaches not only encourage personal development but also enhance overall capacity within the research workforce and public health community. Whether such efforts help to stem “brain drain” is perhaps unknowable. What is clear is that the project has strengthened scientific partnerships, enhanced knowledge, skills and confidence among young researchers, and helped position them and their institutions to be more effective partners in future collaborations. The project also achieved considerable success in building connections between Lao institutions, including the Nutrition Center, the University of Health Sciences, and the Lao Tropical and Public Health Institute. Interaction among these groups was part of the project design, and the opportunity to study together, engage in research together, and travel together resulted in new professional in-country connections and relationships that are mutually reinforcing. Fostering collaboration among multiple stakeholders (multi-sectoral and interdisciplinary) and creating efficient working systems or research ecosystems facilitates knowledge updating, and the sharing of frameworks, results, strategies and lessons learned among stakeholders. Such collaboration can help ensure that various areas of work remain distinct but are shared, responsive to the country’s needs, and beneficial to all partners.

## Figures and Tables

**Fig. 4:**
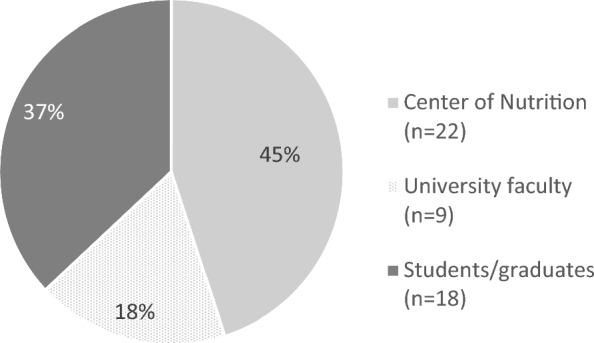
Institutional affiliation of survey respondents

**Fig. 5:**
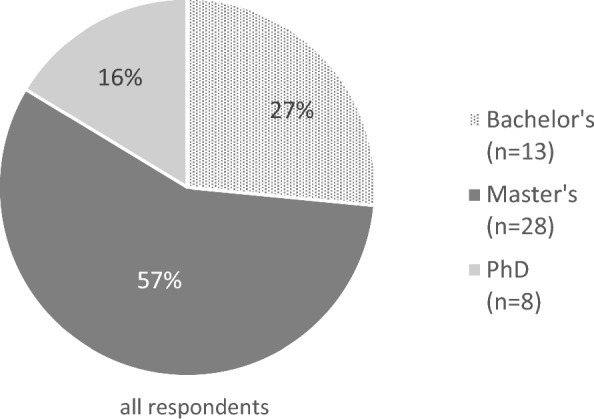
Highest degree earned by survey respondents

**Fig. 6:**
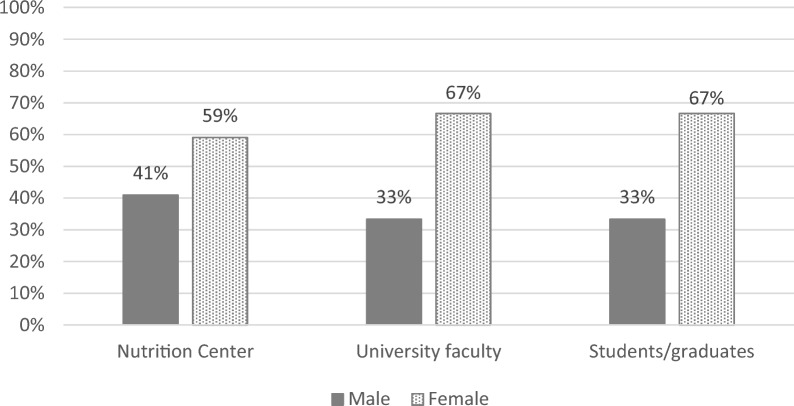
Proportion of survey respondents based on affiliation and gender

**Fig. 7:**
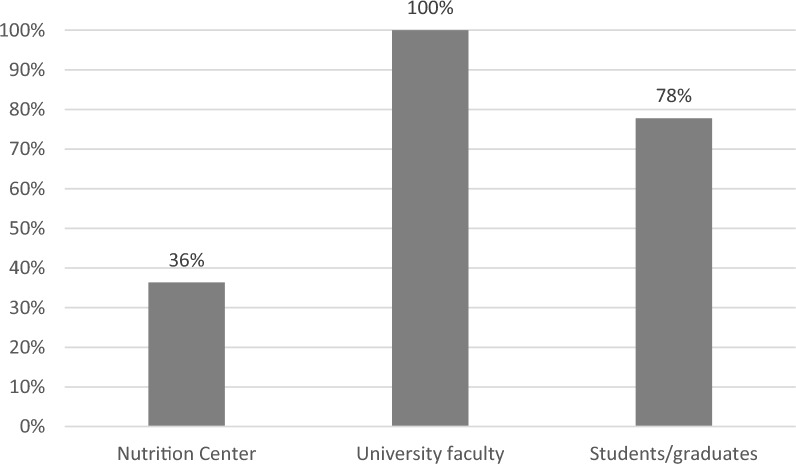
Previous research training of survey respondents

**Fig. 8:**
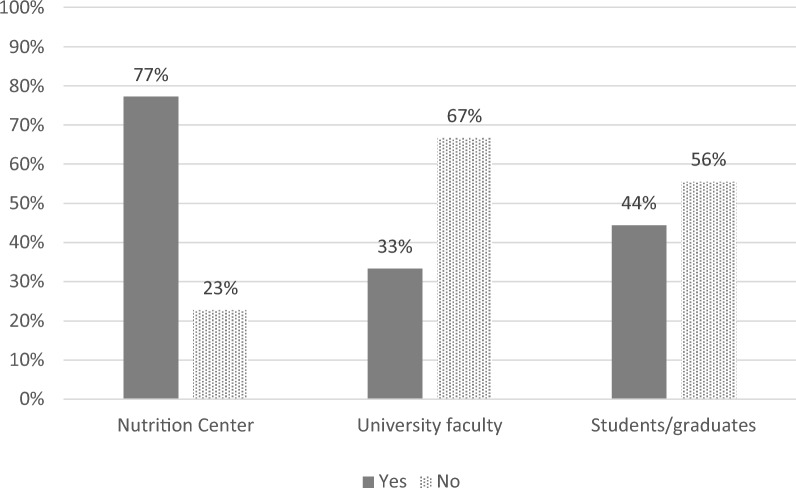
Previous formal courses in nutrition of survey respondents

## Appendix: Characteristics of survey respondents

Figures 4, 5, 6 and 7 display characteristics of respondents to the formal surveys conducted among staff at the Nutrition Center, Lao TPHI and UHS in 2019 and 2020, and with recent graduates of a Netherlands-based training program. These provide context for activities and help to better understand the audience for project training activities. Of 49 participants, 45% were members of the Center staff, 37% were students or recent graduates of the universities included, and 18% were university faculty (Fig. 4).

The most frequent level of formal degree training among respondents (Fig. 5) was a Master’s Degree (57%), followed by a Bachelor’s Degree (27%) and a PhD (16%). PhDs were often found among university faculty, a large proportion of whom received their degree training in Laos.

Roughly two-thirds of respondents were female, with a slightly larger proportion of males at the Center (Fig. 6).

All university faculty reported some prior training in research (Fig. 7), but only one-third of Center staff reported such training. Turning to exposure to nutrition topics, the proportions were reversed: while three-quarters of Center staff reported having taken at least one course in nutrition, only one-third of public health university faculty reported formal training in nutrition—in some cases many years ago and therefore perhaps not providing current knowledge and methods (Fig. 8).

## Footnotes

1 Although planned as a four-year project, the COVID-19 global pandemic delayed and interrupted some Phase 2 and Phase 3 activities. A 1-year no-cost extension from the sponsor and an extension of the MOU by the government of Laos allowed the project to extend work through September 2024.

2 For reasons related to the MOU, project activities were confined to Vientiane and the nearby capital region, and institutions under the purview of the Ministry of Health.

3 Prior to administering the surveys, they were submitted for approval to Purdue University’s Institutional Review Board (IRB) and judged, on the basis of their focus on training needs assessment, to be exempt from requirements of formal research review.

4 The project worked most directly with the MoH, but in a way that left open the possibility of working with other members of the Lao Nutrition Secretariat, including the Ministry of Agriculture and Forestry (MAF), the Ministry of Planning and Investment (MPI), and the Ministry of Education and Sports (MoES). The MPI was viewed at the start as a potentially important allied ministry, as it houses the Lao Statistics Bureau (LSB), but our actual interactions with LSB proved to be limited.

5 Project achievements are grouped into a set of eight activities and briefly described. A project website (https://ag.purdue.edu/department/ipia/anrcb/index.html) provides an archive of the activities and outputs described in this article.
